# Semiclassical and VSCF/VCI Calculations of the Vibrational Energies of *trans*- and *gauche*-Ethanol Using a CCSD(T) Potential Energy Surface

**DOI:** 10.1021/acs.jpca.2c06322

**Published:** 2022-10-14

**Authors:** Riccardo Conte, Apurba Nandi, Chen Qu, Qi Yu, Paul L. Houston, Joel M. Bowman

**Affiliations:** † Dipartimento di Chimica, Università degli Studi di Milano, via Golgi 19, 20133 Milano, Italy; ‡ Department of Chemistry and Cherry L. Emerson Center for Scientific Computation, Emory University, Atlanta, Georgia 30322, United States; § Independent Researcher, Toronto, Ontario M9B0E3, Canada; ∥ Department of Chemistry Yale University, New Haven, Connecticut 06520, United States; ⊥ Department of Chemistry and Chemical Biology, Cornell University, Ithaca, New York 14853, United States; # Department of Chemistry and Biochemistry, Georgia Institute of Technology, Atlanta, Georgia 30332, United States

## Abstract

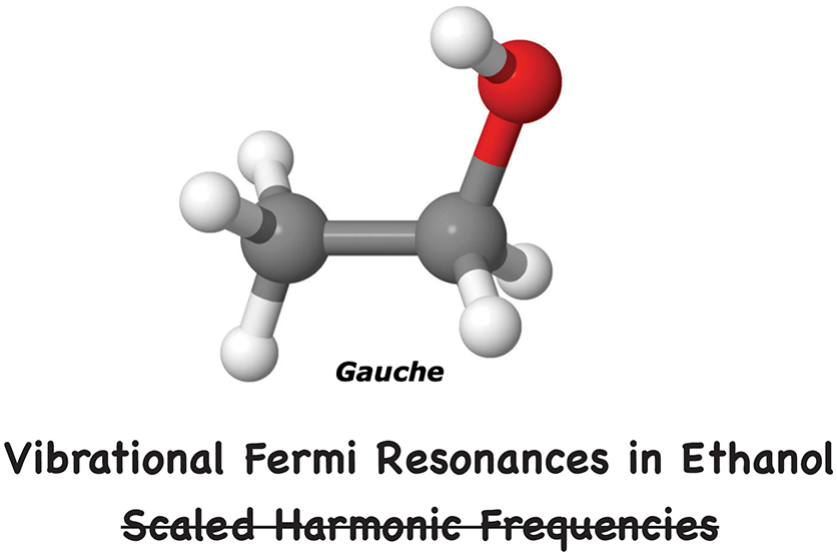

A recent full-dimensional Δ-Machine learning potential energy surface (PES) for ethanol is employed in semiclassical and vibrational self-consistent field (VSCF) and virtual-state configuration interaction (VCI) calculations, using MULTIMODE, to determine the anharmonic vibrational frequencies of vibration for both the *trans* and *gauche* conformers of ethanol. Both semiclassical and VSCF/VCI energies agree well with the experimental data. We find significant mixing between the VSCF basis states due to Fermi resonances between bending and stretching modes. The same effects are also accurately described by the full-dimensional semiclassical calculations. These are the first high-level anharmonic calculations using a PES, in particular a “gold-standard” CCSD(T) one.

## Introduction

From the very early days of quantum mechanics, the holy grail of theoretical chemists has been to put quantum mechanics to use as a computational tool that would some day rival the precision of experiment. The foundational, specific goal has been to develop first-principles potentials, i.e., from quantum mechanics, that govern nuclear motion. Progress in doing this for ever-larger molecules has been dramatic in the past 15 or so years.

Developing high-dimensional, *ab initio*-based potential energy surfaces (PESs) remains an active area of theoretical and computational research. Significant progress has been made in the development of machine learning (ML) approaches to generate potential energy surfaces (PESs) for systems with more than five atoms, based on fitting thousands of CCSD(T) energies.^1−4^ The quantum chemical methods capturing a substantial part of electron correlation, such as coupled-cluster with singles and doubles (CCSD), coupled-cluster with perturbative triples [CCSD(T)], and so on, have a formidable scaling, leading to the requirement of high speed processor, memory, and secondary storage. Thus, there is a bottleneck for developing the PES at high level theory with the increase of molecular size. Due to the steep scaling of the gold standard CCSD(T) theory (∼*N*^7^, *N* being the number of basis functions), it is computationally demanding to build PESs of systems with more than 9–10 atoms. (Many researchers do not consider this number of atoms as a “large molecule”; however, it is used here as a computational boundary for the CCSD(T) method.) Therefore, people are bound to use low-level electronic structure methods such as density functional theory (DFT) and Møller–Plesset second-order perturbation theory (MP2) to generate PESs for large molecules.

The PESs of molecules having more than 10 atoms using CCSD(T) level of theory are generally conspicuous by their absence. One 10-atom PES using the method we are aware of is the formic acid dimer (HCOOH)_2_,^5^ which contains 6 heavy atoms. It was developed by Bowman and co-workers in 2016. This was a major computational effort at the CCSD(T)-F12a/haTZ (VTZ for H and aVTZ for C and O) level of theory, which was a fit to 13475 electronic energies. A 9-atom PES for the chemical reaction Cl + C_2_H_6_ was recently developed by Papp et al. using a composite MP2/CCSD(T) method.^6^ Examples of potentials for 6- and 7-atom chemical reactions which are fits to tens of thousands or even hundreds of thousands of CCSD(T) energies have also been reported.^1,3,4,7,8^

The increasing dimensionality of the PES with the increase in number of atoms requires large training data sets to fit the PES. Thus, given the intense interest and progress in moving to larger molecules and clusters, where high-level methods are prohibitively expensive, the use of lower-level methods such as DFT and MP2 is understandable. These methods also provide analytical gradients, and this is an important source of data needed for larger systems. Our group has made use of the permutationally invariant polynomial (PIP) approach for developing PESs of *N*-methylacetamide,^9,10^ glycine,^11^ and tropolone.^12^

To circumvent this bottleneck, machine learning (ML) approaches are being used to bring a PES based on a low-level of electronic structure theory (DFT or MP2) to a higher level (CCSD(T)) one. There are two popular methods currently being investigated to achieve this goal. One is the “transfer learning” (TL), and the other is the “Δ-machine learning” (Δ-ML).

TL has been developed extensively in the context of artificial neural networks,^13^ and much of the work in that field has been brought into chemistry.^14−18^ The basic idea of TL is that a fit obtained from one source of data (perhaps a large one) can be corrected for a related problem by using limited data and by making hopefully small training alterations to the parameters obtained in the first fit. Therefore, in the present context of PES fitting, a ML-PES fit to low-level electronic energies/gradients can be reused as the starting point of the model for an ML-PES with the accuracy of a high-level electronic structure theory. As noted, this is typically done with artificial neural networks, where weights and biases trained on lower-level data hopefully require minor changes in response to additional training using high-level data. Recently, Meuwly and co-workers applied TL to improve the MP2-based neural network PESs for malonaldehyde, acetoacetaldehyde, and acetylacetone using thousands of local CCSD(T) energies.^18^

The other approach is Δ-machine learning (ML). In this approach a correction is made to a property data set obtained using an efficient, low-level *ab initio* theory such as DFT or MP2.^15−19^ We applied this Δ-ML approach to correct an numbers of PESs based on DFT electronic energies and gradients.^20^ Initially, this was successfully done for CH_4_ and H_3_O^+^ and for 12-atom *N*-methylacetamide.^20^ For *N*-methylacetamide, the PES included both the *cis* and *trans* isomers and the saddle points separating them. More recently, Δ-ML was extensively applied to the 15-atom acetylacetone (AcAc, CH_3_COCH_2_COCH_3_) molecule. This not only developed a full-dimensional PES at CCSD(T) level but also was successfully applied to compute the quantum zero point energy and ground state wave function using diffusion Monte Carlo (DMC) algorithm as well as to determine the tunneling splitting of H-transfer process.^21^ In all cases, a relatively small number of coupled cluster energies were obtained over the same large span of configurations used to get the lower-level DFT PES.

Our most recent application of Δ-ML has been to a full-dimensional PES for the 9-atom ethanol (CH_3_CH_2_OH) molecule at the CCSD(T) level.^22^ Ethanol is widely used as a solvent in chemical reactions, and it has great importance in combustion chemistry. Ethanol is the leading biofuel in the transportation sector, where it is mainly used in a form of reformulated gasoline.^23,24^ Thus, the study of ethanol chemistry in internal combustion engines is of high interest from scientific, industrial, and environmental perspectives. Ethanol exists as a mixture of *trans* or *anti* and *gauche* (±) conformers in both solid, liquid, and gaseous state.^25−27^ It is well-known that the energy gap between the two conformers is quite small; experimentally, it is observed that Δ*G* is 0.12 (0.02) kcal/mol in favor of the *trans* conformer.^26^ Ethanol also has a 3-fold methyl torsional potential which makes its potential surface much more complex. These aspects have been investigated when presenting the new PES. Diffusion Monte Carlo (DMC) calculations performed on the new PES have shown that the global minimum is of the *trans* configuration even when starting from the *gauche* geometry. In this work we complete our study of ethanol by examining the fundamental frequencies of vibration of both conformers, which are expected to be influenced by quantum state mixing and Fermi resonances. To accomplish this task we employ full-dimensional semiclassical (SC) calculations and vibrational self-consistent field (VSCF) and virtual-state configuration interaction (VCI) calculations using MULTIMODE.

The paper is organized as follows. In the next section we briefly describe the PES employed and then provide theoretical and computational details about MULTIMODE and Semiclassical simulations. This is followed by the “Results and Discussion” section in which we compare the calculated frequencies of vibration with experimental data. Finally, the “Summary and Conclusions” section ends the paper.

## Theory and Computational Details

### CCSD(T) PES of Ethanol

The full-dimensional CCSD(T) PES of ethanol used here has been recently reported,^22^ so we give only a brief summary here. The development of this PES can be divided into two parts: low-level DFT PES (*V*_LL_) and a correction PES (Δ*V*_CC–LL_). Initially, a low-level DFT PES is developed for ethanol using the efficient B3LYP/6-311+G(d,p) level of theory, and then a correction is made using a sparse set of a relatively small number of *ab initio* CCSD(T) energies to determine the Δ-ML surface using the simple equation.^20^
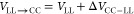
1where *V*_LL→CC_ is the corrected PES, *V*_LL_ is a PES fit to low-level electronic data, and Δ*V*_CC–LL_ is the correction PES based on high-level coupled cluster energies. The assumption underlying the hoped-for small number of high-level energies is that the difference Δ*V*_CC–LL_ is not as strongly varying as *V*_LL_ with respect to nuclear configuration.

This *V*_LL_ PES is a permutationally invariant polynomial fit to 8500 energies and their corresponding gradients at B3LYP/6-311+G(d,p) level of theory spanning the energy range of 0–35 000 cm^–1^. For this fit, we used a maximum polynomial order of 4 with permutational symmetry of 321111, leading to a total of 14752 PIP basis functions and linear coefficients whose values were determined by linear least-squares regression. More details of this PES can be found elsewhere.^28^

To develop the correction PES, we train Δ*V*_CC–LL_ on the difference between the CCSD(T)-F12a/aug-cc-pVDZ and DFT absolute energies for 2069 geometries. A low-order PIP fit was employed because the difference Δ*V*_CC–LL_ is not as strongly varying as *V*_LL_ with respect to the nuclear configuration. We used maximum polynomial order of 2 with permutational symmetry 321111 to fit the training data set which leads to a total of 208 PIP bases. The PIP basis to fit these *V*_LL_ and Δ*V*_CC–LL_ PESs were generated using our latest MSA software.^29,30^ The corrected PES was analyzed and verified previously and already used in unrestricted diffusion Monte Carlo (DMC) and semiclassical calculations to compute accurate zero-point energies (ZPEs) of the *trans* and *gauche* conformers. Details of this PES and analyses can be found in ref (22). Here we use this PES in new vibrational calculations of excited states. These are done using MULTIMODE VSCF/VCI and adiabatically switched (AS) semiclassical initial value representation (SCIVR) methods, which are briefly described next.

### MULTIMODE Calculations

Postharmonic quantum methods based on vibrational self-consistent field (VSCF) and virtual-state configuration interaction (VCI) approaches have been known for almost 50 years. These methods have been implemented in our software called MULTIMODE. First, we present a brief recap of the VSCF^31,32^ and VSCF/VCI scheme^33^ in MULTIMODE.^34−36^ The computational code is based on the rigorous Watson Hamiltonian^37^ in mass-scaled normal coordinates, ***Q***, for nonlinear molecules. This Hamiltonian is given by
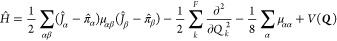
2where α(β) represent the *x*, *y*, *z* coordinates, *Ĵ*_α_ and π̂_α_ are the components of the total and vibrational angular momenta respectively, μ_αβ_ is the inverse of effective moment of inertia tensor, and *V*(***Q***) is the full potential in terms of normal coordinates. The number of normal modes is denoted by *F*, and for nonlinear molecules *F* equals 3*N* – 6. In many applications of this Hamiltonian in the literature, the vibrational angular momentum terms are neglected and this approximation leads to an inaccurate result. Therefore, we include these terms in the MULTIMODE software.

In general there are two major bottlenecks in applications to the VSCF/VCI scheme. One is the numerical evaluation of matrix elements (multidimensional integrals) and the second is the size of the H-matrix. Both naively have exponential dependence on the number of normal coordinates. An effective approach to deal with exponential scaling of matrix elements we represent the full potential in a hierarchical *n*-mode representation (*n*MR).^34^ In normal coordinates, this representation is given by
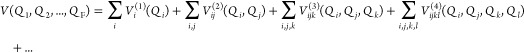
3where *V*_*i*_^(1)^(*Q*_*i*_) is the one-mode potential, i.e., the 1D cut through the full-dimensional PES in each mode, one-by-one, *V*_*ij*_^(2)^(*Q*_*i*_,*Q*_*j*_) is the intrinsic 2-mode potential among all pairs of modes, and so on. Here, intrinsic means that the any *n*-mode term is zero if any of the arguments is zero. Also, each term in the representation is in principle of infinite order in the sense of a Taylor series expansion. So for example, *V*^(1)^(*Q*) might look like a full Morse potential.

This representation has been used for nearly 20 years by a number of research groups; a sample of these are refs (34−36) and (38−41). It continues to be actively used in a variety of applications and theoretical developments.^42−47^ In MULTIMODE the maximum value of *n* is 6. However, from numerous tests it appears that a 4MR typically gives energies that are converged to within roughly 1–5 cm^–1^.^48−50^ Thus, we generally use 4MR with an existing full-dimensional PES and this is also done here.

The second major bottleneck to all VCI calculations is the size of the H-matrix, which as noted already can scale exponentially with the number of vibrational modes. There are many strategies to deal with this. Basically, they all limit the size of the excitation space, with many schemes taken from electronic structure theory. For example, the excitation space can be limited by using the hierarchical scheme of single, double, triple, and further excitations. MULTIMODE uses this among other schemes and can consider up to quintuple excitations. A major difference with electronic structure theory is that the nuclear interactions go beyond two-body. This is immediately clear from the *n*-mode representation. Thus, MULTIMODE tailors the excitation scheme for each term in this representation. Other schemes to prune the CI basis have been suggested, and the reader is referred to reviews^40,42,48,51−56^ for more details.

We note that the above basic VSCF/VCI scheme with the *n*-mode representation has been implemented in Molpro by Rauhut and co-workers with the option to obtain the electronic energies directly on *n*-mode grids, with *n* up to 4 or from an existing potential.^57^ Of course numerous enhancements and modifications to the basic scheme can be found there.

Finally, some comments on the limitations of rectilinear normal modes and thus the Watson Hamiltonian are in order for ethanol, which has large amplitude torsional modes. These are not expected to be accurately described, especially for excited states which will exhibit large amplitude curvilinear motion. We do note that the reaction path version of MULTIMODE^58^ is able to describe these. However, because such motion is not the focus of the present work, we do not use this version, as it is also more computationally demanding than the version we adopt here. Thus, the calculations we present are more reliable quantitatively in the high-frequency region than in the low-frequency region.

### Semiclassical Theory and Calculations

An alternative approach we employ to calculate the anharmonic frequencies is represented by the adiabatically switched (AS) semiclassical initial value representation (SCIVR) technique.^59−61^ AS SCIVR is a recently developed two-step procedure able to describe quantum effects starting from classical trajectories. Therefore, AS SCIVR is a member of the family of semiclassical methods (see, for instance, refs (62−70)), and it features a characteristic way to determine the starting conditions of the semiclassical dynamics. In AS SCIVR, one starts from harmonic quantization and slowly, adiabatically, switches on the actual molecular Hamiltonian. The final geometry and momenta of the adiabatic switching run serve as starting conditions for the subsequent semiclassical dynamics trajectory. This procedure is applied to a distribution of harmonically quantized starting conditions.

The adiabatic switching Hamiltonian is^71−73^
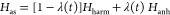
4where λ(*t*) is with switching function
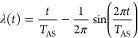
5*H*_harm_ is the harmonic Hamiltonian built from the harmonic frequencies of vibration, and *H*_anh_ is the actual molecular vibrational Hamiltonian. We chose *T*_AS_ equal to 25000 a.u. (about 0.6 ps), and we employed time steps of 10 a.u. for a total of 4400 trajectories.

Once the adiabatic switching run is over, the trajectories are evolved according to *H*_anh_ for another 25000 a.u. with same step size to collect the dynamical data needed for the semiclassical calculation. For this purpose we use Kaledin and Miller’s time-average formula
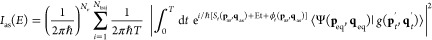
6where *I*_as_(*E*) indicates that a vibrational spectral density is calculated as a function of the vibrational energy *E. I*_as_ is peaked at the eigenvalues of the vibrational Hamiltonian, the lowest one being the ZPE. Frequencies of vibration are obtained by difference between the relevant eigenvalues and the ZPE. In eq 6*N*_v_ is the number of vibrational degrees of freedom of the system, i.e., 21 in the case of ethanol. *T* is the total evolution time of the dynamics for the semiclassical part of the simulation. As anticipated, we chose *T* equal to 25000 a.u. with a time step size of 10 a.u. (**p**_*t*_^′^, **q**_*t*_^′^) is the instantaneous full-dimensional phase space trajectory started at time 0 from the final adiabatic-switching phase space condition (**p**_as_, **q**_as_). *S*_t_ is the classical action along the semiclassical trajectory, and ϕ_*t*_ is the phase of the Herman–Kluk pre-exponential factor based on the elements of the stability matrix and defined as
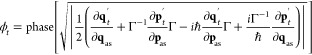
7where Γ is an *N*_*v*_ × *N*_*v*_ matrix usually chosen to be diagonal with elements numerically equal to the harmonic frequencies. Based on Liouville’s theorem, the stability (or monodromy) matrix has the property to have its determinant equal to 1 along the entire trajectory. However, classical chaotic dynamics can lead to numerical inaccuracies in the propagation, so, following a common procedure in semiclassical calculations, we have rejected the trajectories based on a 1% tolerance threshold on the monodromy matrix determinant value. Finally, the working formula is completed by the quantum mechanical overlap between a quantum reference state |Ψ⟩ and a coherent state |*g*⟩ with the following representation in configuration space
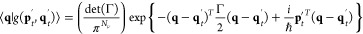
8The reference state |Ψ⟩ is usually chosen to be itself a coherent state. In eq 6 |Ψ⟩ is written as |Ψ(**p**_eq_,**q**_eq_)⟩, where **p**_eq_ stands for the linear momenta obtained in harmonic approximation setting the geometry at the equilibrium one (**q**_eq_).

## Results and Discussion

**Figure 1:**
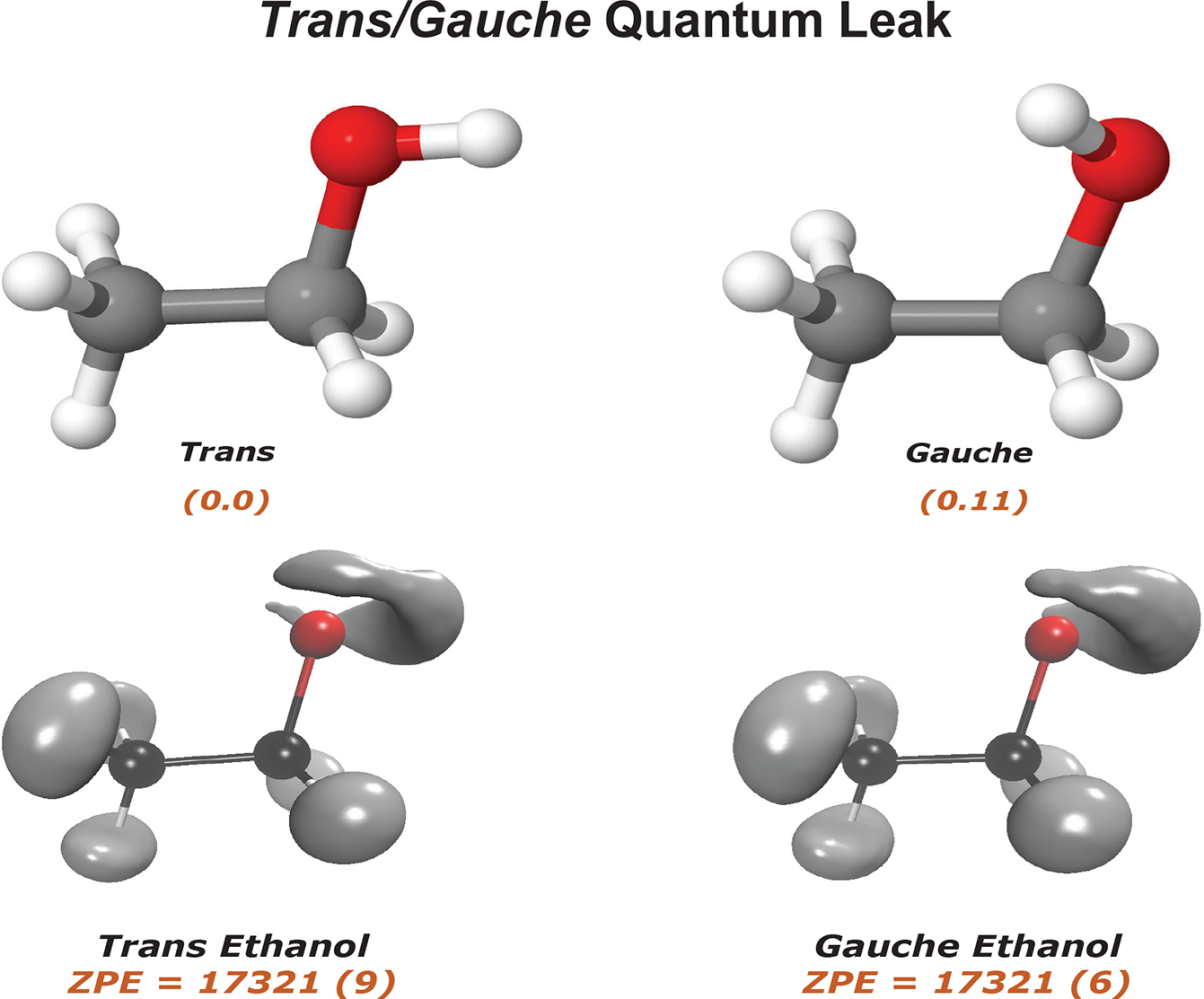
Geometry and ground state wave function for *trans*- and *gauche*-ethanol. Reproduced with permission from ref (22). Copyight 2022 American Chemical Society.

Our CCSD(T)-level PES for ethanol^22^ has been employed for all results presented in this section. The lowest quantum vibrational energy level of a molecule is known as the zero-point energy. A calculation of the ZPE and the associated wave function can be obtained by means of Diffusion Monte Carlo (DMC) calculations. When these are performed for the two ethanol conformers, the same ZPE value is found (17321 cm^–1^), and the wave function reveals the leaky nature of this molecule. As shown in Figure 1, the ground state resembles the *trans* conformer independent of the starting conformer in the DMC calculation, but substantial delocalization to the *gauche* conformer is present.^22^ The leaky nature of the ZPE wave function is responsible for the difficulties to isolate the conformers even at very low temperature, but these results do not preclude concentrated probability density for either conformer for excited states. Indeed, Raman, IR, and spectroscopy experiments implicate the presence of the two conformers.^74^ For instance, a band contour is typical of the signal of the OH stretches and several simplified and reduced-dimensional potentials have been proposed to confirm the experimental assignments.^75,76^ However, these calculations need refinement. Therefore, availability of a full-dimensional and very accurate PES has stimulated us to calculate the fundamental frequencies of vibration for both *trans*- and *gauche*-ethanol.

MULTIMODE calculations are performed using version 5.1.4.^31,34,36^ For all the calculations, a four-mode representation of the potential in mass-scaled normal coordinates and a three-mode representation of the effective inverse moment of inertia for the vibrational angular momentum terms in the exact Watson Hamiltonian are used.^37^ The formalism is based on CI from the virtual space of the ground vibrational state VSCF Hamiltonian. Here we explore reduced-mode coupling models, i.e., 11- and 15-mode models, where these sets of modes start with the highest frequency OH-stretch and proceed in decreasing frequency. In both cases the maximum mode combination excitations are 10 10 10 8, which means that single through triple excitations extend to a maximum sum of quanta of 10, and for quadruple excitations the maximum is 8. This excitation space leads to CI matrix sizes of 45486 and 155026 for 11- and 15-mode calculations, respectively. We compute 200 CI vibrational states up to the energy of 4000 cm^–1^.

AS SCIVR calculations are performed as described in the previous section. The rejection rate due to chaotic trajectories is found to be about 60%. Therefore, calculations are based on about 2000 complete trajectories, which is enough for AS SCIVR to warrant reliability and accuracy of results given the fast convergence of the method.^59^

Rejection is mainly due to the CH_3_ rotor motion. Rejection of these trajectories may lead to some inaccuracies in the SC estimate of rotor motions, but these are low-frequency ones. The reported results demonstrate this effect has no influence on the SC description of higher frequency vibrations.

Tables 1 and 2 show MULTIMODE and semiclassical AS SCIVR anharmonic frequencies with the corresponding experimental and previous scaled harmonic ones.^76,77^ First, note that for many states the 11- and 15-mode calculations are with a few cm^–1^ of each other. For those cases where larger differences are seen, the 15-mode results are closer to experiment. There are more of these instances for *gauche* than *trans*. Also, the same set of states labeled as “mixed” (to be discussed in more detail below) appear in both sets of calculations. Second, there is generally good agreement between MULTIMODE and AS SCIVR frequencies as well as experimental ones. Third, the scaled harmonic results, from two groups,^76,77^ are also in good agreement with the experiment, but this is the result of empirical scaling factors. The scale factors are different and this is because of significant differences in harmonic frequencies. For instance, the harmonic frequencies for the *trans* OH-stretch are, in cm^–1^, 3779(MP2)^77^ and 3756(DFT),^76^ and for the *gauche* OH-stretch they are 3772(MP2)^77^ and 3740(DFT).^76^ None of these agree well with our recently reported corresponding values of 3862(3853) and 3845(3837).^22^ These are from the PES (in parentheses we give the direct calculations at the CCSD(T)-F12/aug-cc-pVDZ level of theory). One more example is for the *trans* CH_2_–symmetric stretch (sstr): 3062(MP2)^77^ and 2926 (DFT).^76^ These differ substantially from the PES and direct CCSD(T) values of 2995 and 3001, respectively.

**Table 1:** Vibrational Frequencies for *trans*-Ethanol^a^

mode	scl-harm (ref (77))^b^	scl-harm (ref (76))^c^	MM (11 modes)	MM (15 modes)	AS SCIVR	expt (ref (77))
7(CO–str)	1090	1093	****	1101	1088	1090 (1093)
8(CH_2_–rck)	1160	1150	****	1160	1148	1166
9(COH–bnd)	1248	1251	****	1251	1242	1241 (1245)
10(CH_2_–twst)	1268	1269	****	1276	1271	1275
11(CH_3_–sdef)	1375	1375	1367	1370	1363	1367
12(CH_2_–wag)	1431	1423	1427	1426	1420	1450 (1430)
13(CH_3_–adef′)	1455	1458	1444	1443	1440	1455
14(CH_3_–adef″)	1472	1476	1459	1459	1456	1480 (1460)
15(CH_2_–sdef)	1503	1500	1491	1489	1481	1500 (1460)
16(CH_2_–sstr)	2872	2865	2810^d^	2811^d^	2881	2888 (2887)
17(CH_2_–astr)	2912	2887	2864^d^	2884^d^	2888	2901
18(CH_3_–sstr)	2926	2941	2939^d^	2937^d^	2933	2922
19(CH_3_–astr″)	3012	3011	2976^d^	2977^d^	2983	2987
20(CH_3_–astr′)	3022	3014	2978^d^	2981^d^	2986	2992
21(OH–str)	3585	3678	3679	3672	3676	3676 (3675)
MAE	13	12	19	13	8	

a Harmonic, MULTIMODE (4MR), AS SCIVR, and experimental IR and Raman gas values. Numbers in parentheses are from Raman experiment. MAE is the mean absolute error with respect to the closest experimental frequency.

b Scaled harmonic at MP2/6-31G(d) level. Scaling factors: 1.0 for heavy atom bend; 0.88 for CH stretches (str) and deformations (def); 0.90 for all other modes.

c Scaled harmonic at DFT/B97-3c level. Scaling factor 0.979.

d These states are mixed; see text and Table 3 for details.

**Table 2:** Vibrational Frequencies for *gauche*-Ethanol^a^

mode	scl-harm (ref (77))^b^	scl-harm (ref (76))^c^	MM (11 modes)	MM (15 modes)	AS SCIVR	expt (ref (77))
7(CO–str)	1063	1064	****	1085	1060	1066
8(CH_2_–rck)	1119	1129	****	1124	1115	1117
9(CH_2_–twst)	1259	1256	****	1259	1247	1249
10(COH–bnd)	1345	1348	****	1337	1332	1342
11(CH_2_–wag)	1373	1376	1370	1370	1369	1373
12(CH_3_–sdef)	1396	1391	1393	1393	1386	1394 (1430)
13(CH_3_–adef′)	1461	1464	1448	1447	1448	1460
14(CH_3_–adef″)	1466	1467	1454	1454	1451	1465 (1460)
15(CH_2_–sdef)	1490	1489	1480	1480	1475	1493 (1460)
16(CH_2_–sstr)	2886	2874	2867^d^	2880^d^	2885	2912
17(CH_3_–sstr)	2912	2924	2874^d^	2923^d^	2909	2936
18(CH_2_–astr)	2978	2965	2927^d^	2950^d^	2957	2972
19(CH_3_–astr′)	2996	2990	2958^d^	2979^d^	2977	2987
20(CH_3_–astr″)	3015	3010	2976^d^	2978^d^	2985	2994
21(OH–str)	3579	3662	3659	3653	3655	3662
MAE	13	8	22	12	10	

a Harmonic, MULTIMODE (4MR), AS SCIVR, and experimental IR gas values. Experimental values in parentheses refer to the Raman experiment. MAE is the mean absolute error with respect to the closest experimental frequency.

b Scaled harmonic at MP2/6-31G(d) level. Scaling factors: 1.0 for heavy atom bend; 0.88 for CH stretches (str) and deformations (def); 0.90 for all other modes.

c Scaled harmonic at DFT/B97-3c level. Scaling factor 0.979.

d These states are mixed ones. See text and Table 4 for details. In the fourth column, the values 2950 for mode 18 and 2979 for mode 19 were employed in the MAE calculation.

**Figure 2:**
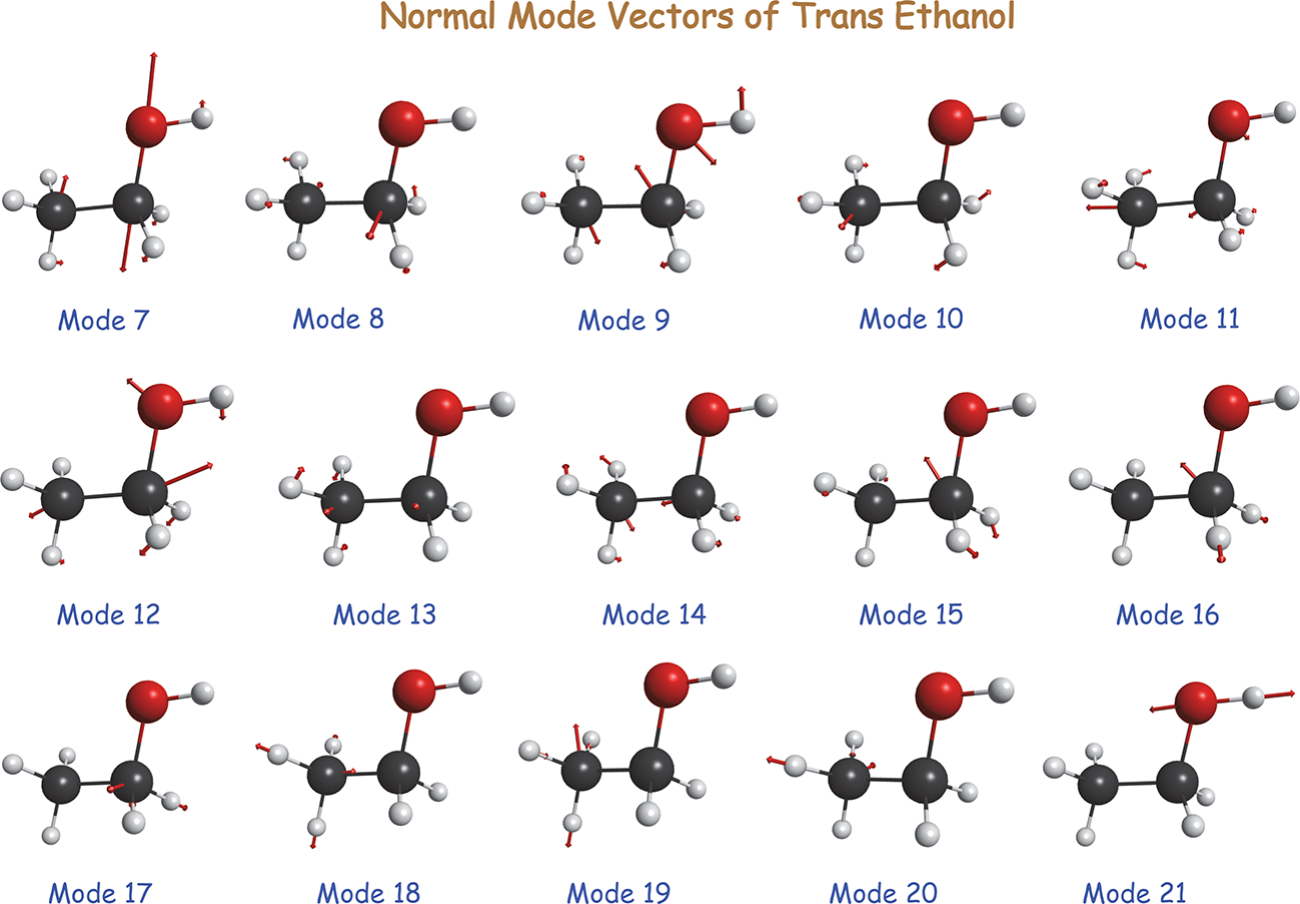
Schematic of normal mode vectors of *trans*-ethanol.

A more general critique of using scaled frequencies in the present case is the significant mixing in a number of CH_2_ and CH_3_ stretches noted in the footnote to these tables. Details of this mixing are given in Tables 3 and 4. There the three largest VCI coefficients in the expansion basis for the 15-mode calculations are given for each “MULTIMODE frequency”, and the normal mode vectors for *trans* ethanol are depicted in Figure 2. The ones for *gauche* ethanol are quite similar. In general the nominal assignment would be based on the largest coefficient. However, as seen there are several states where there are two basis functions with nearly equal coefficients. One example in Table 3 is for the overtone of *trans* CH_2_–wag, denoted 2ν_12_, with the fundamental of *trans* CH_2_–sstr, denoted ν_16_. This is an example of classic 2:1 Fermi resonance, where the corresponding harmonic frequencies (see Table 1 of ref (22)) are close to the 1:2 ratio. The other resonance is with the combination band ν_12_ + ν_11_. Note that the sum of the squares of these coefficients adds to 0.86, so there are in fact other basis functions that make up the remainder of the expansion of this anharmonic eigenstate. It is worth mentioning that we also found similar VCI coefficients for the 11-modes calculations, which demonstrates that these resonances are present in both MULTIMODE calculations. However, for some states the coupling modes are different compared to the 15-mode calculation. This is because the low frequency bending, rocking, and twisting modes are playing an important role in some resonance states in 15-mode calculation, but they are completely missing in the 11-mode calculation.

**Table 3:** Three Largest VSCF/VCI Expansion Coefficients of Indicated Energies (cm^–1^) Frequencies for *trans*-Ethanol

*trans*-ethanol			
MULTIMODE frequency	coupling modes	quanta	coeff.
2811	CH_2_–wag	2ν_12_	0.6051
	CH_2_–sstr	ν_16_	–0.5347
	CH_3_–sdef + CH_2_–wag	ν_11_ + ν_12_	–0.4571
2884	CH_2_–astr	ν_17_	–0.8634
	CH_2_–twst + CH_2_–wag	ν_10_ + ν_12_	0.2818
	CH_2_–rck + CH_2_–sdef	ν_8_ + ν_15_	–0.2263
2937	CH_3_–sstr	ν_18_	0.6277
	CH_3_–adef″	2ν_14_	–0.5442
	CH_3_–adef′	2ν_13_	0.3019
2977	CH_3_–astr″	ν_19_	0.8309
	CH_3_–adef″ + CH_2_–sdef	ν_14_ + ν_15_	–0.3612
	CH_3_–adef″	2ν_14_	–0.2379
2981	CH_3_–astr′	ν_20_	0.9237
	CH_3_–adef′ + CH_3_–adef″	ν_13_ + ν_14_	0.1871
	CH_2_–wag + CH_3_–adef′	ν_12_ + ν_13_	–0.1319

**Table 4:** Three Largest Absolute Magnitude Coupling Coefficients of MULTIMODE Frequencies for *gauche*-Ethanol

*gauche*-ethanol			
MULTIMODE frequency	coupling modes	quanta	coeff.
2880	CH_2_–sstr	ν_16_	0.5852
	CH_3_–sdef + CH_3_–adef″	ν_12_ + ν_14_	–0.4138
	CH_3_–adef′	2ν_13_	0.2968
2923	CH_3_–adef″	2ν_14_	–0.5771
	CH_3_–sstr	ν_17_	–0.4987
	CH_3_–adef′	2ν_13_	–0.3752
2931	CH_2_–astr	ν_18_	–0.5134
	CH_3_–adef′ + CH_2_–sdef	ν_13_ + ν_15_	0.4367
	CH_3_–adef″ + CH_2_–sdef	ν_14_ + ν_15_	0.4249
2950	CH_2_–astr	ν_18_	0.5068
	CH_2_–sdef	2ν_15_	–0.4341
	CH_3_–adef″ + CH_2_–sdef	ν_14_ + ν_15_	0.4097
2959	CH_2_–sdef	2ν_15_	–0.6244
	CH_3_–astr′	ν_19_	–0.4643
	CH_2_–astr	ν_18_	–0.4046
2978	CH_3_–astr″	ν_20_	0.8087
	CH_3_–adef″ + CH_2_–sdef	ν_14_ + ν_15_	0.3754
	CH_3_–astr′	ν_19_	–0.2759
2979	CH_2_–sdef	2ν_15_	0.5336
	CH_3_–astr′	ν_19_	–0.5294
	CH_3_–adef″ + CH_2_–sdef	ν_14_ + ν_15_	0.3885

The presence of mixed states, notably due to Fermi resonances, found with MULTIMODE are evidently not a “problem” for the AS SCIVR simulations. There are two aspects that make AS SCIVR efficient in this task. One is that calculations, trajectories and potential are full-dimensional and this allows one to take into account couplings between all modes. Second, the presence in eq 6 of coherent states, which have a Gaussian shape and therefore a tail in phase space, allows one to correctly collect quantum eigenenergies even if the energy of the trajectories is not perfectly tailored for the state under investigation. Interestingly, the VSCF/VCI energy of mode 16 of *trans*-ethanol, for which the mixing analysis was given above, differs by 70 and 77 cm^–1^ with respect to the AS SCIVR frequency and the experimental frequency, respectively. At this point the source of the difference is not clear. Hopefully, independent quantum calculations can be done to resolve this issue. Unfortunately, our computer resources preclude larger MULTIMODE calculations. Also, the Fermi resonances predicted in the MM and AS SCIVR calculations should result in spectral features that could be observed in high-resolution, ideally very cold, IR spectra. Unfortunately, the present experimental spectra^77^ are not high resolution and they are further complicated by the presence of both conformers.

## Summary and Conclusions

A recently developed, full-dimensional CCSD(T) potential energy surface for ethanol has been employed to compute the anharmonic vibrational frequencies of both *trans*- and *gauche*-ethanol. We found good agreement between MULTIMODE and semiclassical AS SCIVR calculations as well as the previously reported experimental results as shown in Tables 1 and 2. In addition, we also observed significant mixing between the vibrational states and Fermi resonances when low-frequency bending modes are included during VCI calculations. Therefore combination of a precise PES and efficient quantum approximate approaches allows one to study accurately complex effects characterizing vibrational spectra. This work also demonstrates the possibility to perform very accurate quantum simulations when the high-energy region of the potential is adequately sampled. In fact, the AS SCIVR simulations require running classical trajectories at energies even higher than the zero-point one, and we found no issues employing the PES at such high energies.

We found mode 16 of *trans*-ethanol to be strongly mixed, and we got different estimates of the vibrational frequency from Multimode and AS SCIVR calculations. Additional quantum mechanical calculations and even higher resolution experimental spectra could help in clarifying the issue and calculations here presented could serve as benchmark.

A possible future development of this work consists in comparing VCI and SC intensities to the experiment. This will require construction of a dipole moment surface since one is currently not available for ethanol.

Another possible future development of this work concerns the study of the delocalization of excited states. This could be for instance tackled by calculating and examining semiclassical wave functions of the excited states.

## Supporting Information Available

Zip archive containing all files needed to run the ethanol PES employed in this work (ZIP)
